# Editorial: Application of emerging technologies aiming at the recovery of biomolecules

**DOI:** 10.3389/fchem.2025.1557083

**Published:** 2025-02-06

**Authors:** Daniel Lachos Perez, Grazielle Náthia Neves, Wojciech Smulek, Paulo César Torres Mayanga

**Affiliations:** ^1^ Department of Plastics Engineering, University of Massachusetts Lowell, Lowell, MA, United States; ^2^ Research Group for Bioactives -Analysis and Application, National Food Institute, Technical University of Denmark, Lyngby, Denmark; ^3^ Institute of Chemical Technology and Engineering, Poznan University of Technology, Poznan, Poland; ^4^ Innovative Technology, Food and Health Research Group, Departamento de Ingeniería de Alimentos y Productos Agropecuarios, Facultad de Industrias Alimentarias, Universidad Nacional Agraria la Molina, Lima, Peru; ^5^ Instituto de Investigación de Bioquímica y Biología Molecular, Universidad Nacional Agraria La Molina, Lima, Peru

**Keywords:** sustainability, circular economy, advanced technologies, bioactive compounds, natural resources

In the search for sustainable solutions to global challenges such as climate change, food security, and human health, recent research highlights the importance of integrating advanced technologies and eco-efficient approaches to valorize natural resources and by-products. The papers presented in this issue address these needs from innovative perspectives. The first paper highlights how supercritical fluid extraction can transform waste from lucuma production into high-quality oil, reducing environmental impact and leveraging valuable bioactive compounds. This approach is also reflected in the second study, where machine learning optimizes the extraction of lipids from microalgae, opening possibilities for applications in biofuels and nutraceuticals. On the other hand, studies on *C. comosum* and *C. sativus* emphasize the potential of natural products in managing metabolic diseases such as diabetes. The combination of phytochemical analysis, biological assays, and molecular simulations not only validates their traditional uses but also opens avenues for developing therapies based on bioactive compounds. *C. sativum* essential oil serves as another example of how volatile compounds can offer dual solutions: functioning as natural antioxidants and antimicrobial agents, with applications in health and food preservation. Finally, the review on rice residues highlights the potential to convert a massive by-product into valuable fuels and chemicals, aligning with the principles of the circular economy. Taken together, these studies underscore the impact of integrating advanced technologies such as supercritical fluid extraction, machine learning, and *in silico* simulations to harness natural resources more efficiently. By promoting sustainable management, they contribute to innovative solutions addressing the challenges of the 21st century. With a focus on sustainability and health, this research pushes the boundaries of knowledge and underscores the role of science in the transition to a greener and more equitable world.

